# A Seismic Strengthening Technique for Reinforced Concrete Columns Using Sprayed FRP

**DOI:** 10.3390/polym8040107

**Published:** 2016-03-24

**Authors:** Kang Seok Lee, Bang Yeon Lee, Soo Yeon Seo

**Affiliations:** 1 School of Architecture, Chonnam National University, Gwangju 61186, Korea; kslnist@jnu.ac.kr; 2 Departmrnt of Architectural Engineering, Korea National University of Transpotation, Chungju 27469, Korea; syseo@ut.ac.kr

**Keywords:** carbon fiber, concrete columns, epoxy, glass fiber, strengthening, sprayed FRP, vinyl ester

## Abstract

Conventional methods for seismic retrofitting of concrete columns include reinforcement with steel plates or steel frame braces, as well as cross-sectional increments and in-filled walls. However, these methods have some disadvantages, such as the increase in mass and the need for precise construction. Fiber-reinforced polymer (FRP) sheets for seismic strengthening of concrete columns using new light-weight composite materials, such as carbon fiber or glass fiber, have been developed, have excellent durability and performance, and are being widely applied to overcome the shortcomings of conventional seismic strengthening methods. Nonetheless, the FRP-sheet reinforcement method also has some drawbacks, such as the need for prior surface treatment, problems at joints, and relatively expensive material costs. In the current research, the structural and material properties associated with a new method for seismic strengthening of concrete columns using FRP were investigated. The new technique is a sprayed FRP system, achieved by mixing chopped glass and carbon fibers with epoxy and vinyl ester resin in the open air and randomly spraying the resulting mixture onto the uneven surface of the concrete columns. This paper reports on the seismic resistance of reinforced concrete columns controlled by shear strengthening using the sprayed FRP system. Five shear column specimens were designed, and then strengthened with sprayed FRP by using different combinations of short carbon or glass fibers and epoxy or vinyl ester resins. There was also a non-strengthened control specimen. Cyclic loading tests were carried out, and the ultimate load carrying capacity and deformation were investigated, as well as hysteresis in the lateral load-drift relationship. The results showed that shear strengths and deformation capacities of shear columns strengthened using sprayed FRP improved markedly, compared with those of the control column. The spraying FRP technique developed in this study can be practically and effectively used for the seismic strengthening of existing concrete columns.

## 1. Introduction

Rapid progress in seismic design has resulted in new reinforced concrete (RC) buildings with improved prospects of satisfactory behavior during an earthquake. However, innovations in seismic design methodologies have simultaneously created some doubts regarding the adequacy of the seismic behavior of existing RC buildings, as shown by the 1995 Kobe Earthquake in Japan, the 1999 Chi-Chi Earthquake in Taiwan, the 2008 Sichuan Earthquake in China, the 2010 Chile Earthquake, the 2011 Christchurch Earthquake in New Zealand, the 2012 Great East Japan Earthquake, and the 2013 Lushan Earthquake in China.

Over the last two decades, rehabilitation procedures have been promoted, and many seismic strengthening techniques have been developed to improve the seismic performance of existing concrete buildings, especially their columns [1,2,3,4,5]. Conventional methods for seismic retrofitting of concrete columns include reinforcement with steel plates or steel frame braces, as well as cross-sectional increments and in-filled walls. However, these methods have some disadvantages, such as the increase in mass and the requirement for precise construction. Others methods, such as fiber-reinforced polymer (FRP) sheets for seismic strengthening of concrete columns using new light-weight composite materials, including carbon fiber and glass fiber, have excellent durability and performance and have been used widely to overcome the shortcomings of conventional seismic strengthening methods [6,7,8]. Nonetheless, the FRP-sheet reinforcement method still has some drawbacks, such as the need for prior surface treatment, problems at joints, and relatively expensive material costs. Recently, to overcome the weakness of FRP, a new class of cement-based composites was introduced in the civil engineering field [9,10,11,12,13,14,15,16]. Thus, there is a continuing need for the development of new strengthening techniques with better workability and reduced costs for concrete columns.

In the current research, the structural effectiveness of a new type for seismic strengthening of concrete columns with FRP is investigated. The proposed technique is a sprayed FRP system, achieved by mixing chopped glass and carbon fibers with epoxy and vinyl ester resin in the open air and randomly spraying the resulting mixture onto the uneven surface of the concrete columns. There has been little research on sprayed FRP [17,18,19,20,21,22,23]. Furthermore, the use of sprayed FRP for seismic strengthening on columns using epoxy or vinyl ester resins has not been fully investigated.

The main purpose of this study was to develop a new technique for seismic strengthening of existing RC columns. This study first involved tensile testing of the composed material, with the length of the chopped glass and carbon fibers as well as the mix ratio of the fibers, epoxy, and vinyl ester resin, all serving as test variables to determine the optimum properties for sprayed FRP on concrete columns. The optimum sprayed FRP, based on the results of this material testing, was used to strengthen RC columns controlled by shear (shear columns). Five specimens of shear columns were prepared and strengthened by sprayed FRP with different combinations of short carbon or glass fibers and epoxy or vinyl ester resins, including a non-strengthened control specimen. Cyclic loading tests were carried out, and the ultimate shear load carrying capacity and deformation were investigated, as well as hysteresis in the lateral load-drift relationship.

Although vinyl ester resin is generally used for sprayed FRP because it hardens rapidly after being applied, this study considered a mixture of stronger epoxy resin and vinyl ester resin to reduce the viscosity of the spray, resulting in an improvement in the overall workability of the sprayed FRP technique. Material properties and cyclic loading tests were conducted to assess the seismic strengthening performance, as well as the practical design equation, of sprayed FRP on RC columns, and to determine the optimal chopped fiber length and fiber-resin mix ratio to achieve the same strength as one layer of the currently used FRP sheets.

## 2. Material Tests

### 2.1. Test Specimens

**Figure 1:**
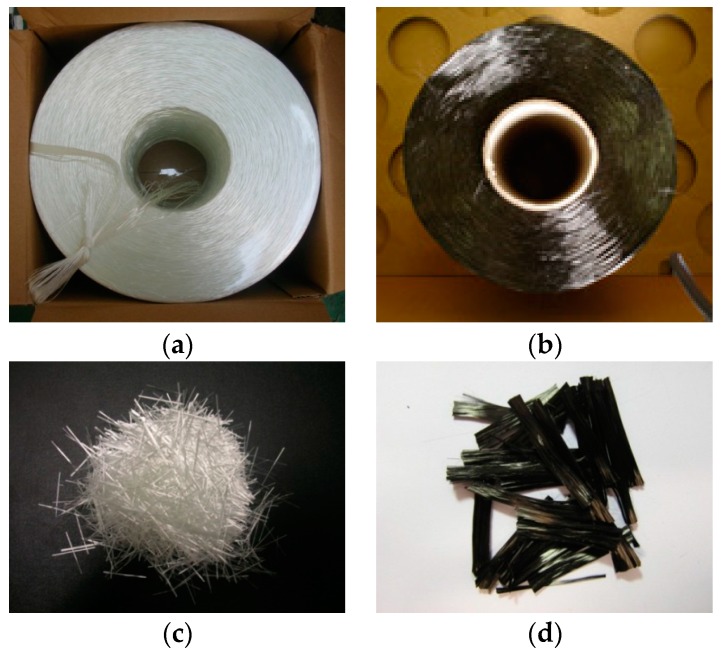
Glass and carbon fibers used in the experiments: (**a**) Glass fiber and (**b**) carbon fiber; (**c**) chopped glass and (**d**) carbon fibers.

**Figure 2:**
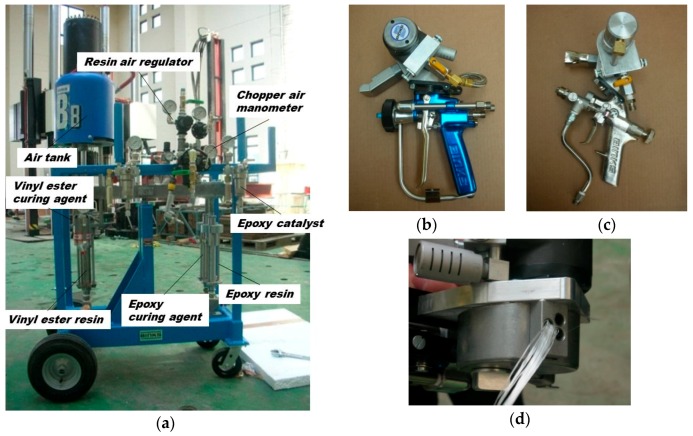
Equipment used for sprayed fiber-reinforced polymer (FRP): (**a**) Spraying equipment; (**b**) Chop-sprayed guns for epoxy and (**c**) for vinyl ester resins; (**d**) Installation of roving type-fiber.

Sprayed FRP is a new research field with a limited body of experimental data, and no standard for FRP material has yet been established. Thus, in this study the existing JIS K7054 [24] specification for tensile testing of glass fiber-reinforced plastic was used. The strengthening material used for the material test included roving-type glass fiber (ERS 2310-233/C; Central Glass Co., Yamaguchi, Japan) [25], roving-type carbon fiber (TR330-50K; Mitsubishi Rayon Co., Tokyo, Japan) [26], as shown in Figure 1, and epoxy and vinyl ester resins (Conclinic Co., Seoul, Korea) [27]. As can be seen in the photograph in Figure 2, spraying equipment, together with guns for chopping carbon and glass fibers (Binks Polycraft, Inc., Franklin Park, IL, USA) [28], were used in the experiments.

In our previous research [23], the material properties for repair and strengthening of RC beams with the sprayed FRP system were investigated. The following material tests in this study were carried out in more detail, based on the tests conducted in the previous research [23].

**Figure 3:**
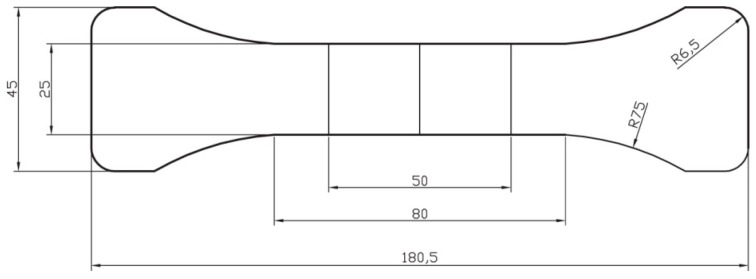
Specimen detail of material test: the unit is mm and thickness is 4 mm.

**Figure 4:**
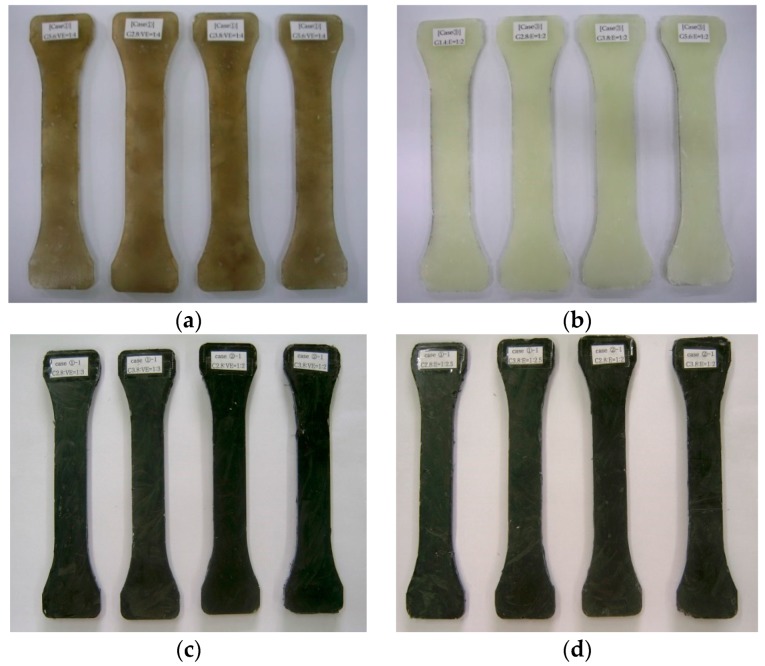
Specimen samples fabricated based on the experimental variables: (**a**) glass fiber + vinyl ester; (**b**) glass fiber + epoxy; (**c**) carbon fiber + vinyl ester; and (**d**) carbon fiber + epoxy specimens.

Experimental variables for the material test were the length of the chopped fibers and the mix ratio of the resin and fibers, which was based on weight. Figure 3 depicts a fiber material specimen for tensile testing, and Table 1 and Table 2 list the material test variables for glass and carbon fibers, respectively. Figure 4 shows specimen samples fabricated based on the experimental variables.

Chopped glass fibers (lengths of 14 and 56 mm) were used as 24 test variables in different mix ratios with vinyl ester or epoxy resins; 120 test specimens having five equal specimens for each variable were fabricated. Eight variables for chopped carbon fibers (lengths of 28 and 38 mm) were set to test, and 40 test specimens having five equal specimens for each variable were fabricated to evaluate the construction workability and performance. In total 160 specimens of glass and carbon fibers were fabricated and tested, respectively.

**Figure 5:**
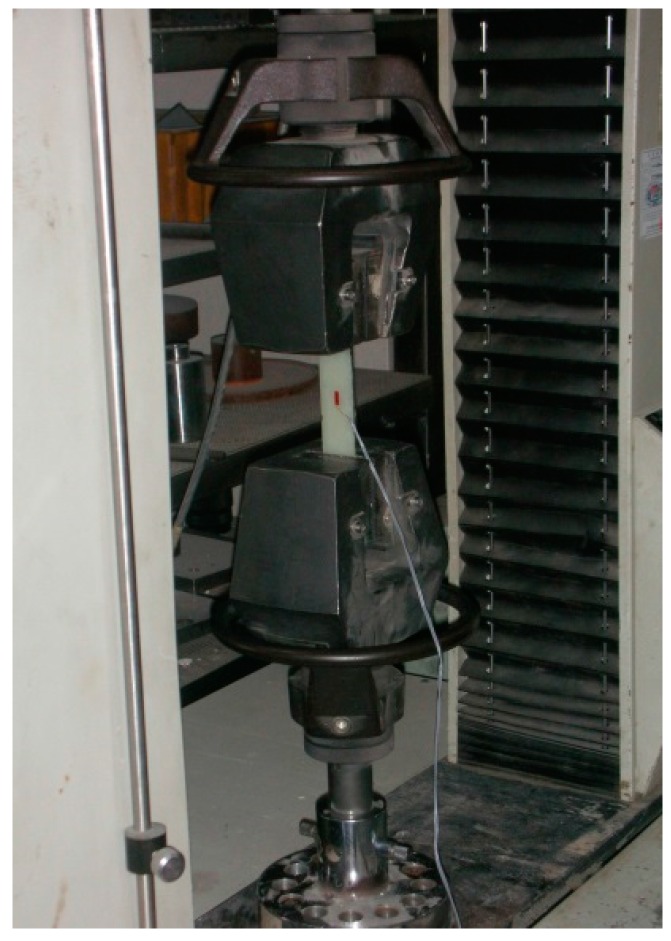
Loading apparatus for the material tests (universal test machine).

The test specimens were cured for 7 days in the open air at 25 °C, after which they were assumed to be completely hard. A strain gauge was installed at the center of each type of test specimen. The tensile stress and strain was measured by a miniature 5-t universal test machine. Figure 5 shows the experimental test setup. The test speed was set to speed type A (1 ± 0.5 mm/min), as specified by JIS K 7054 [24].

### 2.2. Test Results

Tensile strength tests were carried out on five test specimens for each variable. The failure mode in this material test was fracturing at both sides, 40 mm away from the center, and crushing of the joint area. This study used the fracture mode at both sides as the final test result. The results of the material tests, conducted to identify the optimum material properties for the sprayed FRP technique for seismic strengthening, indicated that the tensile strength increased with the length of chopped fibers under the condition that the quantity of the fibers in the mixture was greater than that of the resin. Based on the performance and construction workability of the chopper gun, a fiber length of 38 mm and a resin mix ratio of 1:2 by weight produced the best strength with the least fiber tangling. This material property of the sprayed FRP was the same as estimated in previous research [23].

**Figure 6:**
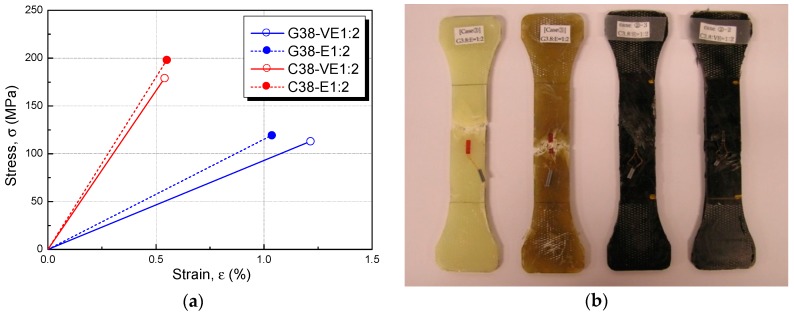
Stress–strain relationships for the optimum mixture and fracture shapes: (**a**) stress–strain relationships of a fiber length of 38 mm and a resin mix ratio of 1:2 in terms of the average value; and (**b**) final fracture shapes.

The stress–strain relationship for the optimum material composition, *i.e.*, that which yielded the best strength, is shown in Figure 6 in terms of the average value, together with the final fracture shapes. The test specimen with chopped glass fiber had a good elastic deformation but was not as strong as that made with chopped carbon fiber. Table 3 lists the results of the material tests and the spray design thickness (*t*_sf_) to be used. The design thickness was calculated from Equation (1) and compared with the properties of the FRP sheets currently used to strengthen existing RC structures in Korea, shown in Table 4, to compute the spray thickness yielding the same tensile strength as one layer of FRP sheet.

(1) $$\frac{\sigma_{t}\left[FRP\;sheet\right]}{\sigma_{t}\left[sprayed\;FRP\right]}\cdot t_{\text{fs}}=t_{\text{sf}}$$

where $\sigma_{t}$ is the tensile strength, $t_{\text{fs}}$ is the construction thickness of the FRP sheet, and $t_{\text{sf}}$ is the design thickness for the sprayed FRP.

## 3. Structure Tests

### 3.1. Specimen Design and Test Variables

**Figure 7:**
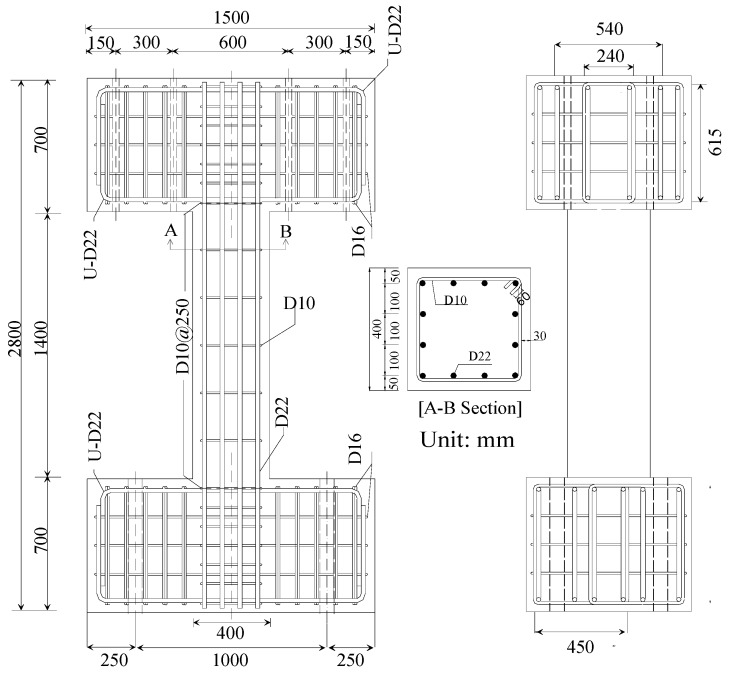
Details of the control specimens (dimensions are in mm).

RC column specimens controlled by shear were designed and fabricated for cyclic loading tests. Figure 7 shows details of the control shear column specimens. The purpose of these tests was to determine the seismic behavior, that is, the ultimate shear and deformation capacities, as well as hysteresis in the lateral load-drift relationship of shear columns, all of which were strengthened with the sprayed FRP technique using the design thickness of material with an equivalent strength of one FRP sheet.

The column specimens were designed according to the guidelines for load-carrying capacity specified by the Japan Building Disaster Prevention Association (JBDPA) [30]. Table 5 gives the specific details of each specimen tested. In total, five test shear failure-type column specimens were prepared. They consisted of a control test specimen (non-strengthened, SC-N), a test specimen strengthened with the sprayed FRP using a glass fiber and vinyl ester resin (SC-S-GV), a test specimen strengthened with a glass fiber and epoxy resin (SC-S-GE), a test specimen strengthened with a carbon fiber and vinyl ester resin (SC-S-CV), and a test specimen strengthened with a carbon fiber and epoxy resin (SC-S-CE).

All specimens had identical dimensions and rebar arrangements. The cross-section of the columns was 400 mm × 400 mm, and the ratio of column clear height to depth (*h*_o_/*D*) = 3.5. Each specimen was prepared with a 12-D22-type SD40 main rebar, reinforced with shear reinforcement D10 steel bars at 250-mm intervals. A sub, with high stiffness, was installed at the top of each specimen to provide confinement for the columns. The average vertical load on columns was ~3 MPa, which is 10% of the nominal compressive strength of the concrete. Table 6 lists the load-carrying capacity, calculated according to the JBDPA [30].

### 3.2. Material Properties of Concrete, Steel Rebar, and Resins

The normal compressive strength of the concrete was *f*_c_ = 30 MPa, and cylindrical compression tests resulted in a compressive strength of 33.0 ± 1.2 MPa. The nominal tensile strength of the steel reinforcing bar (rebar) was 400 MPa. Two different diameter rebars were used: D10 for the shear reinforcement and D22 for the main rebar of the specimens (see Section 3.1 for further details). The uniform building code [31] pertains to RC design in earthquake zones and specifies that the ratio of the tensile stress to the yield stress of the rebar should not be less than 1.25, to ensure adequate ductility under simulated earthquake loading. From tensile testing of the rebar, this ratio was 1.35 for the D10 rebar and 1.28 for the D22 rebar. The tensile strength of the steel rebar was measured using a universal testing machine (UTM); there were obtained 509.9 ± 1.15 MPa for the D10 rebar and 547.6 ± 2.17 MPa for the D22 rebar, where the error margins correspond to the standard deviation of the measurement results.

The sprayed FRP technique involves mixing chopped glass and carbon fibers with epoxy and vinyl ester resin in the open air and randomly spraying the resulting mixture onto the uneven surface of the concrete columns. The strengthening material for the sprayed FRP (Figure 1) was roving-type glass fiber (ERS 2310-233/C; Central Glass Co.) [25], and roving-type carbon fiber (TR330-50K; Mitsubishi Rayon Co.) [26]. Vinyl ester and epoxy resins (Conclinic Co.) [27], with 30 and 45 MPa of flexural strength, were used. Table 7 lists the material properties of the vinyl ester and epoxy resins.

### 3.3. Fabrication of Test Specimens Strengthened by Sprayed FRP

To investigate the seismic resistance, column specimens were designed as controls to exhibit shear failure modes (SC-N). The reinforcement ratios of the SC series were designed so that the structures would exhibit shear failure modes. These structures were then modified, *i.e.*, strengthened using the sprayed FRP, according to different combinations of chopped glass or carbon fibers and vinyl ester or epoxy resins, creating a total of five specimens on which cyclic loading experiments were carried out. All of the specimens had identical dimensions, and a stub with a high stiffness value was installed at the top of each specimen to provide confinement for the columns.

**Figure 8:**
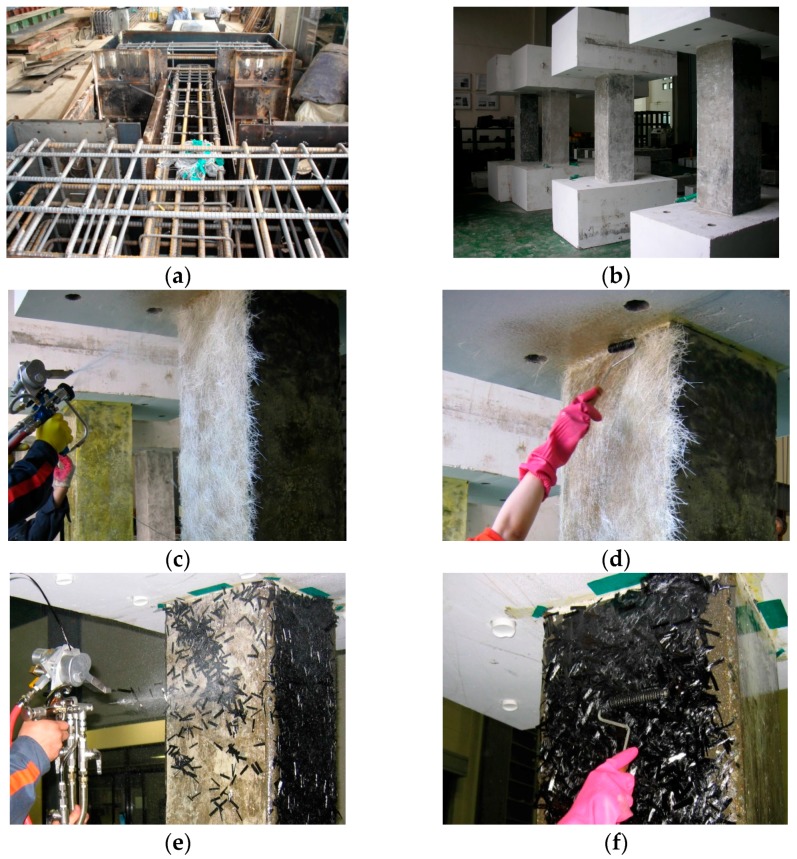
Preparation procedure of the specimens: (**a**) Installation of reinforcing bar; (**b**) control specimens; (**c**) a specimen strengthened using the sprayed FRP with chopped glass fiber; (**d**) surface treatment of sprayed glass fiber; (**e**) a specimen strengthened using the sprayed FRP with chopped carbon fiber; (**f**) surface treatment of sprayed carbon fiber.

Figure 8 illustrates the construction details of the specimens. Following completion of the control specimens, FRP were randomly sprayed on the uneven surface of the concrete columns by mixing chopped glass or carbon fibers with epoxy or vinyl ester resins in the open air using the spraying equipment, as previously shown in Figure 2. The spraying was continued to reach the design thickness calculated in Table 3, which corresponds to the same tensile strength as one layer of FRP sheet, based on the results of material tests. Finally, to enhance the bonding, the sprayed surfaces were treated using a roll-type brush.

### 3.4. Test Procedure

**Figure 9:**
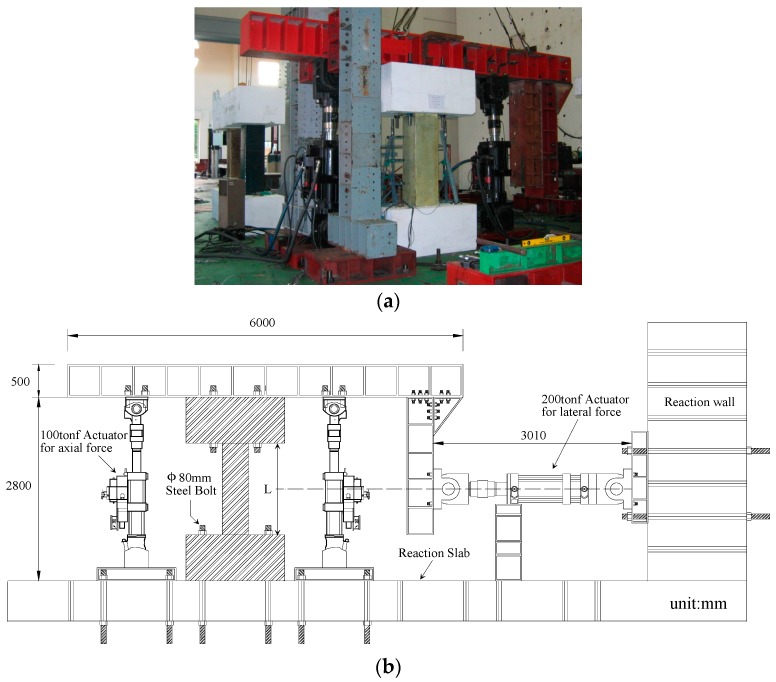
Experimental configuration for the cyclic loading tests: (**a**) photograph and (**b**) diagram views.

The main purpose of the tests was to investigate the seismic resistance of the RC shear columns strengthened using the sprayed FRP system in terms of the maximum load-carrying capacity, deformation, and hysteresis of the lateral load-drift relationship. Figure 9 shows the test set-up for the cyclic loading test. The test set-up was originally developed by the Building Research Institute [32], and has been frequently used to evaluate the seismic behavior of columns. Using this set-up, shear force is effectively applied to the specimens because the action line of lateral load passes the center part of the column specimen by the L-shaped steel frame installed at the top of the specimen. The column was subjected to a constant vertical load of 480 kN during cyclic lateral loading using the two 1000 kN actuators. The vertical load on the column was 3 MPa, which is 10% of the nominal compressive strength of the concrete [32]. A 2000-kN actuator was used to apply the later load.

The load cycles were repeated three times at lateral rotation angles (R) of 0.08%, 0.1%, 0.12%, 0.15%, 0.2%, 0.25%, 0.31%, 0.4%, 0.49%, 1%, 1.24%, 1.54%, 2%, 3%, and 5%. The lateral rotation angle is defined as the relative end displacement at each loading step divided by the clear length of column. Table 8 lists the loading cycles applied to each specimen.

## 4. Failure Sequence and Lateral Load-Drift Curves

The lateral load-carrying capacity of the control and the sprayed FRP-strengthened specimens differed significantly. Generally, all specimens strengthened using the sprayed FRP technique exhibited similar evidence of failure mode, with similar fracture appearance and lateral load-drift relationships. The strengthened specimens, with complete hardening after spraying, showed a shear failure mode, along with the simultaneous movement of the concrete and the reinforcement material, resulting in the eventual fracture of both. In the following discussion, the authors focus on the fracture and failure patterns of the reinforcement materials on the concrete surface in terms of the lateral drift and load-drift relationships during the final stages of the test. Each loading step was identical during the three loading cycles. Table 9 lists the results in terms of maximum shear strength and displacement with positive and negative loads for the five specimens.

### 4.1. Non-Strengthened Control Specimen (SC-N)

**Figure 10:**
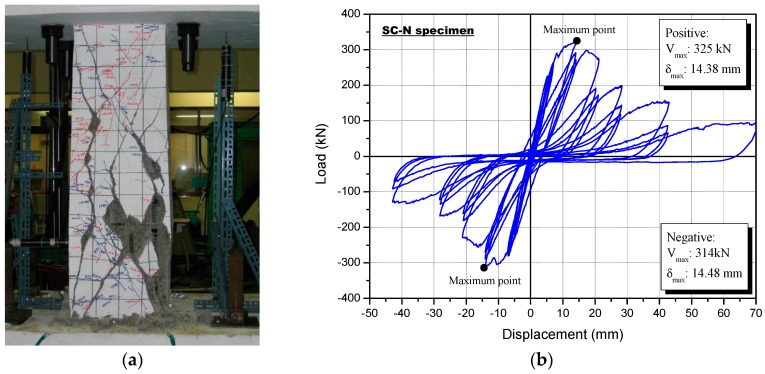
Non-strengthened control (SC-N) specimen following the cyclic loading test: (**a**) failure mode and (**b**) load-displacement relationship.

Figure 10 shows the failure pattern of the SC-N specimen following the final cyclic load test, as well as the lateral load-drift curve for the SC-N specimen, which was designed to exhibit shear failure as shown previously in Table 6. The first observed crack occurred at a negative load of 19.6 kN, and a small flexural crack appeared in the bottom column faces after three cycles at the fourth loading step (*R* = 0.15%). Cracking was not observed in the center of the column. Flexural cracks extended into the middle of the column after step four. Following the seventh loading step (*R* = 0.31%), with a load of both positive 225 kN and negative 219 kN, shear cracks were observed at the top faces of the columns, and diagonal shear cracks appeared, some of which were more than 2 mm wide. When the applied load reached 250 kN, at the ninth positive loading step (*R* = 0.46%), larger shear cracks were observed, with increased widths. During the test, peeling failure due to shear forces from the concrete cover was observed. This was likely the result of insufficient shear confinement.

Shear failure occurred at the bottom of the column following the application of a load of 100 kN, with a lateral drift of 70.0 mm (*R* = 5%). The maximum load capacity of the column of the SC-N specimen was a positive load of 325 kN, with a lateral drift of 14.38 mm (*R* = 1.03%; Table 9). The maximum positive load capacity was similar to the maximum negative load capacity of 314 kN, with a lateral drift of 14.48 mm.

### 4.2. Sprayed FRP-Strengthened Specimens Using Glass Fiber and Vinyl Ester Resin (SC-S-GV)

**Figure 11:**
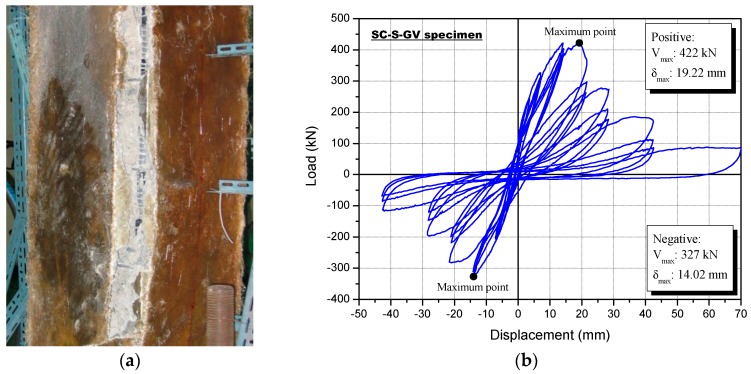
Specimen strengthened with the sprayed FRP using a glass fiber and vinyl ester resin (SC-S-GV) following the cyclic loading test: (**a**) failure mode and (**b**) load-displacement relationship.

Figure 11a shows a photograph of the SC-S-GV specimen following the cyclic loading test, and Figure 11b shows the lateral load-drift curves. The SC-S-GV specimen did not show surface cracks, because the surface had the reinforcement material sprayed on it, with glass fiber and vinyl ester resin. For the first time, a fracturing sound of the glass fiber in the inner part of reinforcement material occurred in the top and bottom column faces following the first cycle of the tenth loading step (*R* = 1%). At a load of 303.8 kN, with a positive loading after step 10, the specimen started fracturing after the simultaneous movement of the reinforcement and the concrete at the edge of the bottom column face, some of which were ~50 mm wide, resulting in eventual debonding.

The maximum load capacity of the SC-S-GV specimen was a positive load of 422 kN, with a lateral drift of 19.22 mm (*R* = 1.37%; Table 9, Figure 11). It should be noted that the concrete and the reinforcement behaved together with complete hardening. The sprayed FRP system for the RC column was therefore an effective reinforcement technique that could markedly increase the shear strength.

### 4.3. Sprayed FRP-Strengthened Specimen Using Glass Fiber and Epoxy Resin (SC-S-GE)

**Figure 12:**
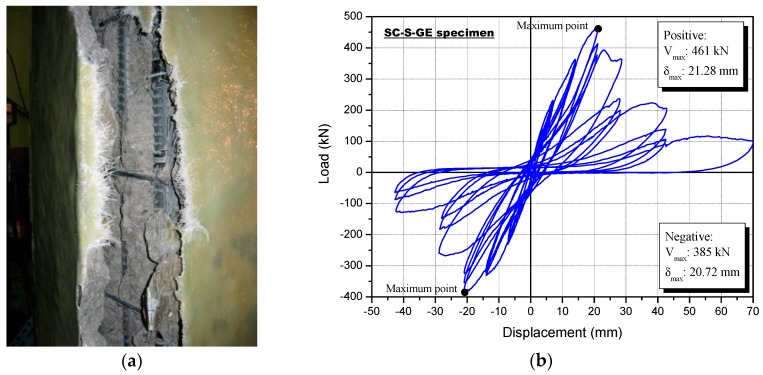
Specimen strengthened with the sprayed FRP using a glass fiber and epoxy resin (SC-S-GE): (**a**) failure mode and (**b**) load-displacement relationship.

The SC-S-GE specimen was strengthened using the sprayed FRP with glass fiber and epoxy resin. Surface cracks were not seen because of the externally sprayed glass fiber and epoxy resin. Figure 12a shows the failure mode of the SC-S-GE specimen following the cyclic loading test, and Figure 12b shows the lateral load-displacement curve. The lateral strength of the SC-S-GE specimen did not increase at the 34th positive loading cycle (12th loading step, *R* = 1.54%) with a maximum load of 461 kN.

Similar to the SC-S-GV specimen, a fracturing sound of the glass fiber in the inner part of the reinforcement material occurred in the top and bottom column faces following the first negative loading cycle of the 10th loading step (*R* = 1%). At a load of 323.4 kN, with a negative loading after step 10, the specimen started fracturing at the edge in the middle of the column. When the applied displacement reached 23 mm, after the 12th positive loading step (*R* = 1.54%), larger fractures were observed, some of which were ~70 mm wide, and the fractures spread to the lower and upper ends of the column specimen.

The concrete and the reinforcement, with complete hardening after spraying, behaved together in the SC-S-GE column test specimen, as shown in Figure 12a, resulting in eventual fracture of both at the 14th step (*R* = 3%).

### 4.4. Sprayed FRP-Strengthened Specimen Using Carbon Fiber and Vinyl Resin (SC-S-CV)

**Figure 13:**
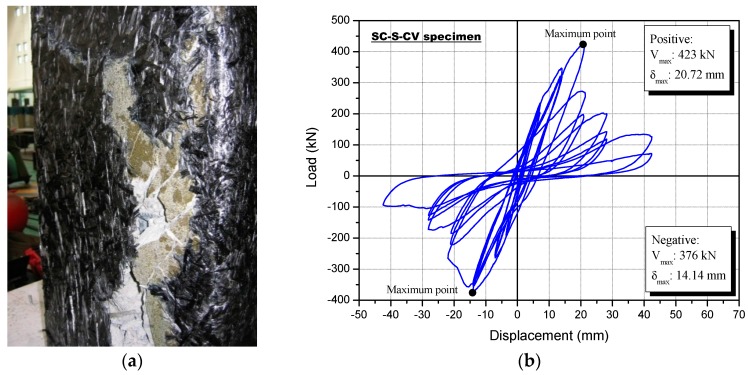
Specimen strengthened with the sprayed FRP using a carbon fiber and vinyl ester resin (SC-S-CV) following the cyclic loading test: (**a**) failure mode and (**b**) load-displacement relationship.

Figure 13 shows the failure mode after the cyclic loading test, as well as the lateral load-displacement curve for the SC-S-CV specimen. The SC-S-CV specimen was reinforced using carbon fiber and vinyl ester resin. Surface cracks were not seen because of the sprayed materials; first, a fracturing sound of the carbon fiber occurred in the top and bottom column faces at the first cycle of the ninth loading step (*R* = 0.49%) with a load of 274 kN.

At a load of 390 kN, with a positive loading after step 11, the specimen started fracturing at the edge of the bottom column faces. After the 12th positive loading step (*R* = 1.54%), larger fractures were observed and they spread to the middle of the column, showing simultaneous movement of the reinforcement and the concrete. Finally, at step 14, the reinforcement fractured and completely debonded at the edge of the lower end of the column specimen. The lateral strength of the SC-S-CV specimen did not increase at the 34th positive loading cycle (12th loading step, *R* = 1.54%) with a maximum load of 423 kN.

### 4.5. Sprayed FRP-Strengthened Specimen Using Carbon Fiber and Epoxy Resin (SC-S-CE)

**Figure 14:**
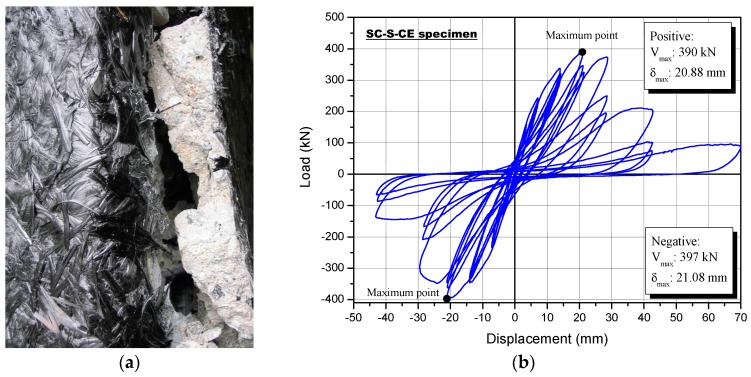
Specimen strengthened with the sprayed FRP using a carbon fiber and epoxy resin (SC-S-CE): (**a**) failure mode and (**b**) load-displacement relationship.

The SC-S-CE specimen was strengthened using the sprayed FRP with carbon fiber and epoxy resin. Surface cracks were not seen because of the externally sprayed carbon fiber and epoxy resin. Figure 14 shows the failure mode following the cyclic loading test, as well as the lateral load-displacement curve. The lateral strength of the SC-S-CE specimen did not increase at the 34th negative loading cycle (12th loading step, *R* = 1.54%) with a load of 397 kN, which showed the maximum load.

A fracturing sound of the glass fiber in the inner part of the reinforcement material occurred in the top and bottom column faces at the first negative loading cycle of the 10th loading step (*R* = 1%) with a load of 333.2 kN.

After the load reached 333.2 kN, the specimen started fracturing at the edge of the middle of the column. When the applied displacement reached 28 mm, after the 12th positive loading step (*R* = 2%), larger fractures were observed, some of which were ~90 mm wide, and the fractures spread to the lower and upper ends of the column specimen.

Similar to the other three strengthened specimens described above, the concrete and the reinforcement, with complete hardening after spraying, behaved together in the SC-S-CE column specimen, as shown in Figure 14a, resulting in eventual fracture of both at the 15th step (*R* = 5%).

### 4.6. Strength and Deformation

**Figure 15:**
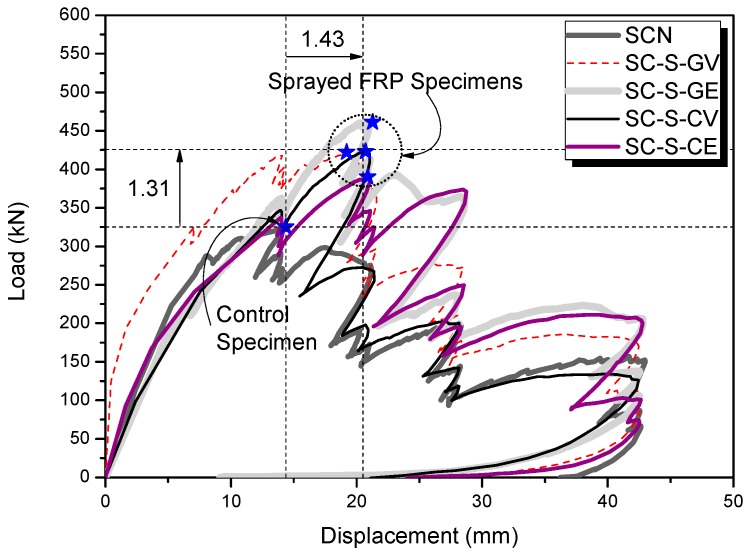
Envelope of the load-displacement relations of the specimens.

Figure 15 shows positive envelope curves of the lateral load-displacement relationship up to *R* = 3% for the sprayed FRP-strengthened specimens (SC-S-GV, SC-S-GE, SC-S-CV, and SC-S-CE) with the non-strengthened control SC-N specimen for comparison with the strengthened specimens. Table 10 lists maximum strength and deformation capacities. The larger of the maximum positive and negative load values were used here (see Figure 10, Figure 11, Figure 12, Figure 13 and Figure 14). The strength ratio (SR) is defined as the ratio of maximum load *V*_max_ of specimens strengthened with the sprayed FRP to that of the SC-N control specimen, and the displacement ratio (DR) indicates the ratio of displacement at the maximum point δ_max_ of the specimens strengthened with the sprayed FRP to that of the SC-N control specimen.

The maximum shear strength of specimens SC-S-GV was 422 kN, that of SC-S-GE was 461 kN, that of SC-S-CV was 423 kN, and that of SC-S-CE was 397 kN; these represent an increase of a factor of ~1.22 to 1.42 (*i.e.*, 22%–42% larger) relative to the SC-N control specimen, where the maximum shear strength was 325 kN. Also, as illustrated in Figure 15, the specimens strengthened using the sprayed FRP exhibited a reinforcement effect, in terms of shear strength, on average 1.31 times greater than the control specimen. The displacement at the maximum strength point (δ_max_) of the four specimens strengthened using the sprayed FRP ranged from ~19.22 to 21.28 mm; this indicates a reinforcement effect (*i.e.*, deformation) on average 1.43 times larger than the SC-N control specimen (Figure 15).

The results regarding the strength and deformation capacities mentioned above show that the sprayed FRP technique for RC columns controlled by shear was an effective retrofitting technique to provide both increased strength and deformation, which is a useful approach for existing low- to medium-rise RC buildings that were not designed and built to seismic specifications. Adequate strength and deformation can reduce the inelastic earthquake response in terms of hysteretic energy dissipation [33,34].

## 5. Applicability of Shear Strengthening Design Equations for the FRP Sheet to Sprayed FRP

In Japan, the current standard equations for calculating the maximum shear strength capacity according to the shear strengthening effects of the reinforcement materials (FRP sheet) include an equation proposed by the JBDPA [35] (Equation (2)), and one specified by the Architectural Institute of Japan (AIJ) [36] (Equation (3)), revised using the shear capacity equation for the truss-arch mechanism on the basis of the effective strain of the FRP sheet based on a regression analysis of existing experimental data. For practical use of FRP sheets according to Equation (3), especially, the AIJ proposed a coefficient of shear strength reduction of α = 0.67, based on a regression analysis of existing experimental data.

Both Equations (2) and (3) were modified by converting a parameter showing shear reinforcing bars, when the FRP sheet reinforcement was considered, in the existing equations to calculate the ultimate shear strength of the columns.

(2) $$V_{u}=\left\{\frac{0.053P_{t}^{0.23}\left(17.6+f_{ck}\right)}{\frac{M}{Q\times d}+0.12}+0.845\sqrt{P_{sw}\times \sigma_{sw}+\alpha \times P_{fw}\times \sigma_{fw}}+0.1\sigma_{0}\right\}\times b\times j$$

where

*P*_t_ = tensile reinforcement ratio (percent)
*f*_ck_ = compressive strength of concrete (N/mm^2^)
*M/Q* = shear span length; default value = *h*_o_/2
*h*_o_ = clear height
*d* = effective depth of the column
  *P*_sw_ = shear reinforcement ratio of shear reinforcing bars
  σ_sw_ = yield strength of the shear reinforcing bars (N/mm^2^)
  α = coefficient of shear strength reduction
  *P*_fw_ = shear reinforcement ratio of FRP sheets
  σ_sw_ = tensile strength of FRP sheets (N/mm^2^)
  σ_0_ = axial stress in the column (N/mm^2^)
*b* = column width (mm)
*j* = distance between the centroids of the tension and compression forces
  (3) $$V_{u}=b\times j_{t}\times \left(P_{sw}\times \sigma_{sw}+\alpha \times P_{fw}\times \sigma_{fw}\right)\times cot\phi +tan\theta \cdot \left(1-\beta \right)\times \upsilon \times f_{ck}\times b\times D/2$$

where

$j_{t}=\;$ distance between the centroids of the main reinforcing bars
$cot\phi :\text{min}\left\{2.0,j_{t}/\left(D\times tan\theta \right),\sqrt{\upsilon \times \frac{f_{\text{ck}}}{P_{\text{sw}}\times \sigma_{\text{sw}}+\alpha \times P_{\text{fw}}\times \sigma_{\text{fw}}}-1.0}\right\}$
ϕ = compressive inner angle of concrete in truss mechanism
  *D* = column depth
$tan\theta =\sqrt{{\left(h_{0}/D\right)}^{2}+1}-h_{0}/D$
υ: effective coefficient of compressive strength of concrete ($\upsilon =0.7-f_{\text{ck}}/200$)
$\beta =\;\left\{\left(1+cot^{2}\phi \right)\cdot \left(P_{\text{sw}}\times \sigma_{\text{sw}}+\alpha \times P_{fw}\times \sigma_{\text{fw}}\right)\right\}/\left(\upsilon \times f_{\text{ck}}\right)$

Kang [37] conducted a comparison study of theoretical values computed from existing equations, expressed in Equations (2) and (3), and experimental test values of column specimens reinforced using FRP sheets. The results indicated that the equation proposed by the JBDPA [35] having a coefficient of strength reduction of α = 1.0 was the most reliable with an average comparison correlation of 0.85 (standard deviation of 0.09), compared with the AIJ equation calculated using a coefficient of strength reduction of α = 0.67.

This study proposes coefficients of shear strength reduction (α), using Equations (4) and (5) of the sprayed FRP strengthening technique based on a comparison between theoretical values calculated from the two existing equations and experimental test results of the column specimens strengthened using the sprayed FRP.

(4) $$\alpha_{1}=\frac{{\left[\left\{\frac{V_{\text{test}}}{b\times j}-\frac{0.053P_{t}^{0.23}\left(17.6+f_{\text{ck}}\right)}{\frac{M}{Q\times d}+0.12}-0.1\sigma_{0}\right\}/0.845\right]}^{2}-P_{\text{sw}}\times \sigma_{\text{sw}}}{P_{\text{fw}}\times \sigma_{\text{fw}}}$$

where

$\alpha_{1}$ = coefficient of shear strength reduction based on Equation (2), specified by the JBDPA [35].

$V_{test}$ = experimental test results of the column specimens strengthened using sprayed FRP (Table 9).

(5) $$\alpha_{2}=\frac{\left\{\frac{V_{\text{test}}-tan\theta \times \left(1-\beta \right)\times \upsilon \times f_{\text{ck}}\times b\times D/2}{b\times j_{t}\times cot\phi }-P_{\text{sw}}\times \sigma_{\text{sw}}\right\}}{P_{\text{fw}}\times \sigma_{\text{fw}}}$$

where

$\alpha_{2}$ = coefficient of shear strength reduction based on Equation (3), modified by the AIJ [36], using the arch-truss mechanism equation for calculating shear strength capacity.

To use Equations (4) and (5) with sprayed FRP, the properties of the mixture of chopped fibers and resins was taken into account using the value of reinforcement design thickness ($t_{\text{sf}}$) for $P_{\text{fw}}$ and the reinforcement strength obtained from the material test ($\sigma_{t}$) for $\sigma_{\text{fw}}$ (Table 3), respectively. When the shear strengthening capacity of sprayed FRP was computed under these conditions without considering the coefficient of shear strength reduction, the shear capacity could not be estimated appropriately because the computation is based on the assumption that the sprayed FRP behaves in tandem with the member.

Because the actual behavior of sprayed FRP results in fracturing and debonding failure at the region of maximum load, there is a need for a coefficient of shear strength reduction of the design strength for sprayed FRP, just as there is for the existing FRP sheet strengthening method [35,36,38]. Thus, the experimental values of Table 10 and the computed values from Equations (2) and (3) were compared to propose a coefficient of shear strength reduction (α_1_ and α_2_) for sprayed FRP, as shown in Equations (4) and (5).

Table 11 lists coefficients of shear strength reduction (α_1_) calculated using Equation (4)—which was derived using Equation (2), proposed by the JBDPA [35]—and those ($\alpha_{2}$) calculated using Equation (5), which was derived based on Equation (3), proposed by the AIJ [36]. As indicated in Table 11, the coefficients of strength reduction (α_1_) for the sprayed FRP system in Equation (4) (JBDPA) were distributed in the range of 0.93–2.25, and the average value was 1.51. The coefficients of strength reduction (α_2_) in Equation (5) (AIJ) had a lower limit value of 0.35, an upper limit value of 0.48, and an average value of 0.41.

**Figure 16:**
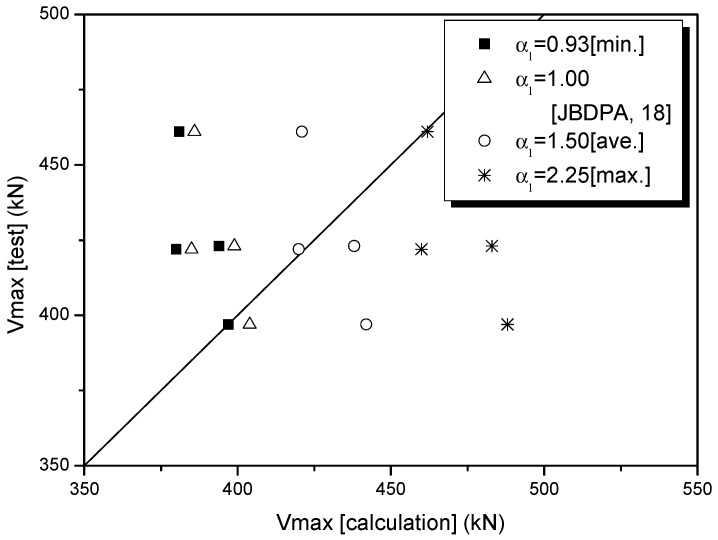
Comparison of *V*_max_ (test) and *V*_max_ (calculation) based on coefficients of strength reduction ($\alpha_{1}$).

**Figure 17:**
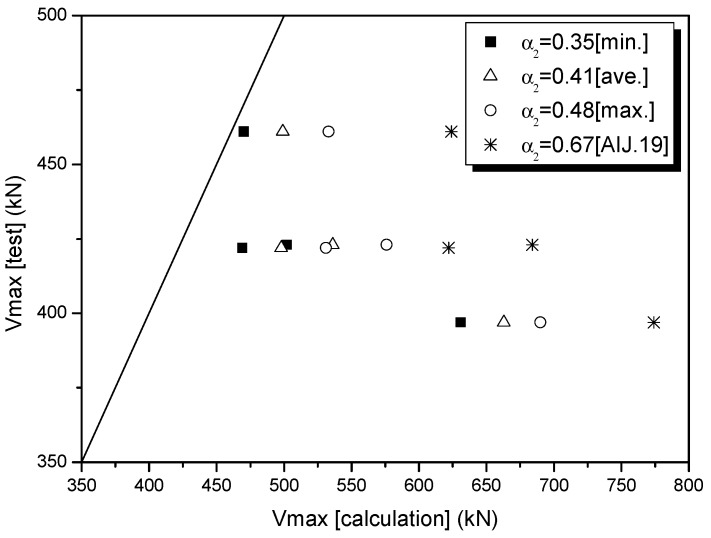
Comparison of *V*_max_ (test) and *V*_max_ (calculation) based on coefficients of strength reduction ($\alpha_{2}$).

Table 12 and Figure 16 show the relationship between the test values of *V*_max_ (test) and the theoretical values of *V*_max_ (calculation), which were computed by applying each of the values for α_1_ (JBDPA), the proposed coefficient of shear strength reduction in Table 11: α_1_ = 1.51 (average α_1_ value); the minimum value of α_1_ = 0.93, and the maximum value of α_1_ = 2.25, together with α_1_ = 1.0, as proposed by Kang [37]. Table 13 and Figure 17 show the relationship between the test values of *V*_max_ (test) and the theoretical values of *V*_max_ (calculation), which were computed by applying each of the values for α_2_ (AIJ), the proposed coefficient of shear strength reduction in Table 11: α_2_ = 0.41 (average α_2_ value); the minimum value of α_2_ = 0.35, and the maximum value of α_2_ = 0.48, together with α_2_ = 0.67, as proposed by the AIJ [36].

Test results using the sprayed FRP technique against shear failure in columns indicated that the coefficient of shear strength reduction (α_2_) in the existing equation specified by the AIJ [36] tended to over-estimate the value of *V*_max_ (test) compared with α_1_ (JBDPA) [35]. Considering the safety of seismic capacity for the shear strengthening of columns, in the sprayed FRP retrofitting method, the shear strength equation calculated using α_1_ = 0.93 (specified by the JBDPA) is the most practical theoretical equation.

## 6. Concluding Remarks

Material tests and structural tests were conducted in this study to determine the optimum properties of sprayed FRP materials for construction workability and field applicability. The material property values to achieve strength equivalent to one layer of existing FRP sheet were determined by the material tests. The results of the material tests were used in cyclic loading structural tests on shear column specimens to investigate the seismic strengthening performance of sprayed FRP, including the maximum load-carrying capacity, deformation, and hysteresis of the lateral load-drift relationship.

Finally, the possibility of using the existing FRP sheet strengthening design equations for sprayed FRP calculations was investigated, and a seismic strengthening design equation for sprayed FRP reinforcement was proposed. The results of this study are summarized below.

(1) The optimum material was found to be 38-mm chopped glass and carbon fibers mixed with resin in a ratio of 1:2. The optimum design thickness for sprayed FRP was 4.4 mm, 4.2 mm and 4.0 mm respectively for chopped glass fiber, vinyl ester resin, and epoxy resin, 3.0 mm for chopped carbon fiber.
(2) The maximum shear strength of specimens strengthened using the sprayed FRP exhibited a reinforcement effect, in terms of shear strength, on average 1.31 (*i.e.*, 31% larger) times greater than the control specimen. The displacement at the maximum strength point of four specimens strengthened using sprayed FRP ranged from ~19.22 to 21.28 mm; this indicates a reinforcement effect (*i.e.*, deformation) on average 1.43 times larger than the control specimen.
(3) Existing FRP sheet design equations are applicable to column test specimens. Considering the safety of seismic capacity for the shear strengthening of columns, in the sprayed FRP retrofitting method the shear strength equation calculating using α_1_ = 0.93, as specified by the JBDPA, is the most practical theoretical equation.
(4) The proposed sprayed FRP technique for RC columns controlled by shear is an effective retrofitting technique providing both increased strength and deformation, which is a useful approach for existing low- to medium-rise RC buildings that are not designed according to the seismic specifications. For further research, the influence of discrepancies between the design thickness and the actual construction thickness and debonding mechanism of the strengthening material and concrete should be examined.

## Figures and Tables

**Table 1:** Material test variables for glass fiber.

Specimen	Materials	Length (mm)	Ratio of mixture (Weight)	Fiber (g)	Resin (g)
G14:VE = 1:4	Glass fiber + Vinyl ester	14	1:4	5.66	22.63
G28:VE = 1:4		28	1:4	5.66	22.63
G38:VE = 1:4		38	1:4	5.66	22.63
G56:VE = 1:4		56	1:4	5.66	22.63
G14:VE = 1:3		14	1:3	7.30	21.89
G28:VE = 1:3		28	1:3	7.30	21.89
G38:VE = 1:3		38	1:3	7.30	21.89
G56:VE = 1:3		56	1:3	7.30	21.89
G14:VE = 1:2		14	1:2	10.27	20.54
G28:VE = 1:2		28	1:2	10.27	20.54
G38:VE = 1:2		38	1:2	10.27	20.54
G56:VE = 1:2		56	1:2	10.27	20.54
G14:E = 1:3.0	Glass fiber + Epoxy	14	1:3	7.35	22
G28:E = 1:3.0		28	1:3	7.35	22
G38:E = 1:3.0		38	1:3	7.35	22
G56:E = 1:3.0		56	1:3	7.35	22
G14:E = 1:2.5		14	1:2.5	8.60	21.5
G28:E = 1:2.5		28	1:2.5	8.60	21.5
G38:E = 1:2.5		38	1:2.5	8.60	21.5
G56:E = 1:2.5		56	1:2.5	8.60	21.5
G14:E = 1:2.0		14	1:2	10.35	20.7
G28:E = 1:2.0		28	1:2	10.35	20.7
G38:E = 1:2.0		38	1:2	10.35	20.7
G56:E = 1:2.0		56	1:2	10.35	20.7

**Table 2:** Material test variables for carbon fiber.

Specimen	Materials	Length (mm)	Ratio of mixture (Weight)	Fiber (g)	Resin (g)
C28:VE = 1:3	Carbon fiber + Vinyl ester	28	1:3	6.97	20.90
C38:VE = 1:3		38	1:3	6.97	20.90
C28:VE = 1:2		28	1:2	9.63	19.26
C38:VE = 1:2		38	1:2	9.63	19.26
C28:E = 1:2.5	Carbon fiber + Epoxy	28	1:2.5	8.14	20.36
C38:E = 1:2.5		38	1:2.5	8.14	20.36
C28:E = 1:2		28	1:2	9.70	19.39
C38:E = 1:2		38	1:2	9.70	19.39

**Table 3:** Test results of the specimens including sprayed fiber-reinforced polymer (FRP) design thickness.

Specimen	Fiber	Resin	Strain *, ε_t_ (%)	Stress *, σ_t_ (MPa)	Design thickness (mm)
G38:VE = 1:2	Glass	VE	1.215	113.15	4.4
G38:E = 1:2	Glass	Epoxy	1.036	119.31	4.2
C38:VE = 1:2	Carbon	VE	0.540	179.1	3.3
C38:E = 1:2	Carbon	Epoxy	0.550	198.1	3.0

* indicates the average value.

**Table 4:** Material properties of the FRP sheets used to strengthen existing concrete structures in Korea.

Material		Tensile strength (MPa)	Modulus of elasticity (MPa)	Construction thickness currently used to strengthen RC structure in Korea (mm)
FRP sheet type	Glass fiber (CAF GL1000; Conclinic) [27]	500	2.5 × 10^4^	1
	Carbon fiber (SK-N300; SK Chemicals) [29]	3,550	2.35 × 10^5^	0.167

**Table 5:** Summary of the specimens.

Specimens	Column clear height *h*_o_ (mm)	Column depth *D* (mm)	*h*_o_/*D*	Tensile reinforcement ratio ρ_f_ (%)	Shear reinforcement ratio ρ_s_ (%)	Strengthening types	
						Fiber type	Resin type
SC-N	1,400	400	3.5	0.97	0.14	–	–
SC-S-GV	1,400	400	3.5	0.97	0.14	Glass	Vinyl ester
SC-S-GE	1400	400	3.5	0.97	0.14	Glass	Epoxy
SC-S-CV	1,400	400	3.5	0.97	0.14	Carbon	vinyl ester
SC-S-CE	1,400	400	3.5	0.97	0.14	Carbon	Epoxy

**Table 6:** Load-carrying capacity of the columns calculated according to JBDPA [30].

Specimens	Axial force *N* (kN)	Ultimate flexural strength *M*u (kN·m)	Shear force at ultimate flexural failure *V*_mu_ (kN)	Ultimate shear strength *V*_su_ (kN)	Ultimate lateral load-carrying capacity *V*_u_ (kN)
SC-N	480.0	284.6	406.6	280.0	280.0

**Table 7:** Material properties of the vinyl ester and epoxy resins.

Classification	Flexural strength (MPa)	Compressive strength (MPa)	Hardening time (h)	Viscosity (cps)	Density (g/cm^3^)
Vinyl ester resin	30	90	24	250	1.04
Epoxy resin	45	100	24	630	1.10

**Table 8:** Loading cycles.

Loading step	1	2	3	4	5	6	7	8
Loading cycles	1–3	4-6	7–9	10–12	13–15	16–18	19–21	22–24
Drift angle (*R*) (%)	0.08	0.1	0.12	0.15	0.2	0.25	0.31	0.4
Lateral drift δ (mm)	1.12	1.4	1.68	2.1	2.8	3.5	4.34	5.6
Loading step	9	10	11	12	13	14	15	–
Loading cycles	25–27	28–30	31–33	34–36	37–39	40–42	43–45	–
Drift angle (*R*) (%)	0.49	1	1.24	1.54	2	3	5	–
Lateral drift δ (mm)	6.86	14.0	17.36	21.56	28.0	42.42	70	–

**Table 9:** The maximum strengths and drifts of the specimens.

Specimen	Positive		Negative		Failure mode
	*V*_max_^a^ (kN)	δ_max_^b^ (mm)	*V*_max_^a^ (kN)	δ_max_^b^ (mm)	
SC-N	325	14.38	314	14.48	Shear failure and collapse of the column
SC-S-GV	422	19.22	327	14.02	The simultaneous behavior of the concrete and the reinforcement material resulted in the eventual fracture of both
SC-S-GE	461	21.28	385	20.72	
SC-S-CV	423	20.72	376	14.14	
SC-S-CE	390	20.88	397	21.08	

Abbreviations: same as Table 5. ^a^ *V*_max_, maximum shear strength; ^b^ δ_max_, drift at the maximum point.

**Table 10:** Summary of the strengths and deformation capacities of the test specimens.

Specimen	Maximum shear strength *V*_max_ (kN)	Displacement at the maximum Point δ_max_ (mm)	Strength ratio (SR)	Displacement ratio (DR)
SC-N	325	14.38	1.00 (325/325)	1.00 (14.38/14.38)
SC-S-GV	422 (30%)	19.22	1.30 (422/325)	1.34 (19.22/14.38)
SC-S-GE	461 (42%)	21.28	1.42 (461/325)	1.48 (21.28/14.38)
SC-S-CV	423 (30%)	20.72	1.30 (423/325)	1.44 (20.72/14.38)
SC-S-CE	397 (22%)	21.08	1.22 (397/325)	1.47 (21.08/14.38)

**Table 11:** Proposed coefficients of strength reduction.

Specimen	Coefficients of strength reduction	
	$\alpha_{1}$ (Equation (4)) (Japan Building Disaster Prevention Association) [35]	$\alpha_{2}$ (Equation (5)) (Architectural Institute of Japan; modified based on the Arch–Truss mechanism equation) [36]
SC-N	–	–
SC-S-GV	1.54	0.41
SC-S-GE	2.25	0.48
SC-S-CV	1.31	0.38
SC-S-CE	0.93	0.35
Average	1.51	0.41

**Table 12:** Comparison of *V*_max_ (test) and *V*_max_ (calculation) based on coefficients of strength reduction.

Specimen	*V*_max_ [Test] (kN)	*V*_max_ [Calculation] (the JBDPA) [35] (kN)			
		$\alpha_{1}=1.0$ (Kang [37])	$\alpha_{1}=0.93$ (Minimum)	$\alpha_{1}=1.5$ (Average)	$\alpha_{1}=2.25$ (Maximum)
SC-N	325	280			
SC-S-GV	422	385	380	420	460
SC-S-GE	461	386	381	421	462
SC-S-CV	423	399	394	438	483
SC-S-CE	397	404	397	442	488

**Table 13:** Comparison of *V*_max_ (test) and *V*_max_ (calculation) based on coefficients of strength reduction.

Specimen	*V*_max_ [test] (kN)	*V*_max_ [Calculation] (the AIJ Modified Based on the Arch–Truss mechanism equation) [36] (kN)			
		$\alpha_{2}=0.35$ (Minimum)	$\alpha_{2}=0.41$ (Average)	$\alpha_{2}=0.48$ (Maximum)	$\alpha_{2}=\;$0.67 (AIJ [35])
SC-N	325	303			
SC-S-GV	422	469	498	531	622
SC-S-GE	461	470	499	533	624
SC-S-CV	423	502	536	576	684
SC-S-CE	397	631	663	690	774
